# Infant feeding and child health and survival in Derbyshire in the early twentieth century^☆^

**DOI:** 10.1016/j.wsif.2016.10.011

**Published:** 2017

**Authors:** Alice Reid

**Affiliations:** The Cambridge Group for the History of Population and Social Structure, Department of Geography, University of Cambridge, Downing Place, Cambridge CB2 3EN, UK

## Abstract

This paper uses detailed records relating to feeding and health for a large sample of infants born in Derbyshire in the early twentieth century to provide a more detailed and nuanced picture than has previously been possible of the extent and duration of breast-feeding, reasons for ceasing to feed and the dangers of feeding in the early twentieth century. Results indicate that breast-feeding was the norm among working class British women in the early twentieth century, but the social gradient was the inverse to that found in Britain today. However this disguises much individual variation and early weaning was more common among twins, illegitimate infants, first births, and women in poor health, which placed infants at greater risk of death from many causes of death, but particularly gastro-intestinal infections. There is evidence that health visitors were successful both in promoting breast-feeding and in supporting safe hand-feeding.

## Introduction

Recent feminist discourses and discussions of biopower portray pro-breast-feeding policies as instruments of gendered social control, embodying deep-seated assumptions about femininity and masculinity (Wolf, 2011, Blum, 1999, Carter, 1995, Wall, 2001). These policies are seen as instruments which enable institutions to police the bodies and behaviour of women, particularly those whose mothering practices may be different to those of the white, middle-class hegemonic group. The language used by these scholars carries the suggestion that such policies are inherently sinister and aimed at surveillance and normalisation, and some authors claim that they can be actively detrimental. For example Millard argues that the detailed advice on how to carry out breast-feeding actually tends to undermine the practice, although she still sees it as beneficial (Millard, 1990). Wolf goes so far as to challenge the orthodoxy that breast-feeding is better for women and children than bottle-feeding, asserting that a neo-liberal culture of risk and personal responsibility forces a mother's own needs to be trumped by what might produce a better (but unproven) outcome for her child (Wolf, 2011). Wall argues that the way that breast-feeding is socially framed suggests that it is possible for all women to do it successfully, engendering intolerance and a lack of support for those for whom it is not possible or who choose not to (Wall, 2001).

Most of these arguments are made in the context of the late twentieth and early twenty-first centuries: in particular, Wolf's claims about the scientifically unproven nature of the superiority of breast-milk is restricted to circumstances where nutritionally appropriate formula milk is available, clean water and high standards of sanitation are provided, and the disease environment is benign. Nevertheless, similar arguments have been made about the infant and child welfare movement in the UK which gathered force in the early twentieth century and included the promotion of breast-feeding and the simultaneous instruction of new mothers in infant care and hygiene as part of the strategy to reduce infant mortality rates. This maternal education or ‘mothercraft’ strategy, fuelled by post-Boer War concerns about ‘national efficiency’, has been condemned by historians as a means of social control, tainted by nineteenth century doctrines which regarded the home as the proper place for women (Lewis, 1980, Dyhouse, 1978, Apple, 1987, Apple, 1995, Davin, 1978). Davin asserts that women were morally blackmailed to conform to expectations: ‘failure to breastfeed… [was a sign ] of maternal irresponsibility, and infant sickness and death could always be explained in such terms’ (Davin, 1978, 13–14). Moore takes a Foucauldian perspective in which the infant welfare movement is viewed as a biopolitical tool of government, a view which chimes with Davin's feminist viewpoint, and which depicts women as singled out as a ‘threat to the population’ and subject to corrective inspection in order to train them to ‘adhere to a pre-existing norm’ which was exemplified by middle-class behaviour (Moore, 2013, 56, 64). However, in a detailed study of child welfare in three different areas, Sian Pooley has found the thrust of ideology and the resulting tenor of the services to have differed between different places (Pooley, 2010). Bromley, in Greater London, conformed to the models depicted by Davin and Moore: middle-class philanthropists striving for national improvement of the working classes. In contrast welfare provision in the Lancashire textile town of Burnley was backed by a rhetoric of civil pride rather than the imperialist agenda, and individual measures were practical and pragmatic, facilitating safe bottle-feeding and the possibility of women returning to work after childbirth.

This paper aims to dig below the rhetoric of social control and blame, to investigate how mothers negotiated the advice from and monitoring by health visitors in the early twentieth century, whether this was benign or sinister. It is focussed on infant feeding, which was one of the main planks of the infant welfare movement. It uses detailed records relating to feeding and health for a large sample of infants born in Derbyshire in the early twentieth century to provide a more detailed and nuanced picture than has previously been possible of the extent and duration of breast-feeding, reasons for ceasing to feed and the dangers of supplementation and artificial-feeding in the early twentieth century. In the analysis in this paper breast-feeding refers to exclusive breast-feeding, artificial- or hand-feeding to a complete absence of breast-milk, and mixed-feeding to both breast-milk and other nutrition. The paper also examines the work of health visitors in relation to infant feeding, concentrating more on whether they had any effects on feeding methods and survival than on their motives.

## Infant feeding in the past and the establishment of health visiting services

For the period leading up to the mid-nineteenth century, estimates of the prevalence of breast and artificial-feeding have been based on sketches in works of fiction and on information in medical journals (Phillips, 1978). Fildes (1986, 352–65) compared recommendations on the length of time to breast-feed with the actual experience of a small sample of real infants based on letters, diaries and case histories, finding the median length of breast-feeding to be around 16 months, around the same or slightly less than that recommended by physicians. However it is difficult to know how representative was this tiny sample which comprised 42 children over 300 years, mainly drawn from the upper classes and including a good proportion of royal infants. Significantly more is known about the early twentieth century, when routine data started to be collected by women sanitary inspectors, health visitors, and infant welfare clinics, initiatives which developed under the Infant Welfare movement (Dwork, 1987, Marks, 1996).

One of the earliest examples of such data was a study read before the Derby Medical Society in April 1905 and subsequently published in the Lancet (Howarth, 1905). William Howarth, the Medical Officer of Health (MOH) for Derby, reported on a local scheme which had started in 1900, whereby registered births were passed to the MOH on a weekly basis. Women inspectors visited infants to provide advice and also collected data on feeding, and the MOH traced deaths in the first year of life. Howarth used data for infants born between November 1900 and November 1903 for the analysis shown in his paper. He found that 63% of infants were breast-fed, 20% hand-fed, and 17% fed by both methods (either sequentially or simultaneously) ‘from a very early stage of their existence’ (Howarth, 1905, 211). He also showed that, at 198 deaths per 1000 infants, mortality was considerably higher among hand-fed infants than among breast- and mixed-fed infants, among whom mortality was 70 and 99 per 1000 respectively. A number of roughly contemporaneous studies in the UK and in North America used similar methods to follow the survival of children fed by different methods, and showed conclusively that artificially-fed children were at significantly greater risk of death than breast-fed (Armstrong, 1904, Davis, 1913, Woodbury, 1922).

Howarth's women inspectors investigated the food given to hand-fed infants, and he concluded that sweetened condensed milk was perhaps the worst food for infants, followed by bread, rusks and other bread-based concoctions. Howarth also noted that patented infant foods varied considerably in quality and their nutritional suitability for infants. He considered contamination of milk to be an important factor, suspecting that proximity to privy middens (toilet systems consisting of a seat above a pit) might be to blame, along with maternal ignorance about matters regarding hygiene. He argued that his system of informing the MOH about new births, which were then visited by women inspectors, was key to the improvement of infant care:

"Although the education of girls at school in the subjects of domestic economy and home nursing would be of the greatest value, a very great deal more can be done by instructing the young mother at home as soon as possible after the baby has made its appearance. To do this would necessitate the notification of every birth to the sanitary authority and visitation by a properly qualified person. Such information is received in this town and visits are made with, I believe, the greatest advantage to both mother and child." Howarth (1905, 213)

Only two years after the publication of Howarth's paper, the Notification of Births Act of 1907 enabled local authorities to establish the sort of system he was advocating (Dwork, 1987, 139; Marland, 1993). It had already been compulsory, since 1837, for parents to register the birth of each child to the local registrar, but they were given a leisurely six weeks to do so. Howarth, and others concerned about the need for supervision and instruction of new mothers, were aware that the first few weeks of an infant's life were by far the most dangerous, and that it might be too late for many mothers and children if they only received help and instruction after the birth was registered. The Notification of Births Act was therefore designed to allow for the visiting of infants in the first few weeks of life. Those local authorities which adopted it required all births to be notified by the attending midwife, doctor, or other attendant, to the local Medical Officer of Health within 36 hours of the birth. The 1907 Act was permissive, meaning that it was up to each local authority to adopt it or not as they chose, but it was held to be a success and was followed eleven years later by the Notification of Births Act 1918 which made the procedures compulsory (Dwork, 1987, 139). The notification of births was generally accompanied by the establishment of a fleet of health visitors (whom Howarth might have called ‘properly qualified persons’) whose job it was to follow up the notified births with visits.

The merits of early visiting by a woman inspector or health visitor are still debated (see Reid, 2001a, 119–20 for an overview), but the notification of births and the health visiting system also allowed monitoring of the sort that enabled Howarth to perform his analysis, and many Medical Officers of Health began to publish feeding statistics in their annual reports. The analyses of such reports form the bulk of what is known about infant feeding in the late nineteenth and early twentieth centuries: Valerie Fildes collected statistics for 22 Local Authorities and 23 London Boroughs for the period 1900–1919, and Peter Atkins' data set includes information from 95 Local Authorities and 28 London Boroughs between 1902 and 1938 (Fildes, 1990, Fildes, 1992, Fildes, 1998, Atkins, 2003). These studies confirm that the majority of infants were breast-fed during their first two months and that hand-feeding was associated with a lethal penalty of high infant mortality (see also Buchanan, 1985, 156; Dyhouse, 1978, 255; Lewis, 1980, 71; Marks, 1996, 107–10).

Although of immense importance in establishing geographic and temporal similarities and differences in feeding patterns and penalties, such studies are not without their limitations. To some extent this is due to the lack of any standard data collection or methodology on the part of those who collected this data in the late nineteenth and early twentieth centuries. There was great variety in the age at which information was collected for the infants, so the ages for which feeding was recorded differed from place to place, hampering comparability. Sometimes the authors of the original reports were unclear about the ages to which their data referred; for example Howarth states that his feeding data refer to ‘a very early stage of infants' existence’ but does not say what that age was (Howarth, 1905, 211). Often the information collected was different and in particular different studies used different definitions of breast- and hand-feeding. Population coverage was different too: some Medical Officers of Health collected information from all women and some only the working classes and it is difficult to tell how representative and comparable were the samples chosen (see Peretz, (1992) for variations in health visiting programmes). It is very difficult to control for the different proportions visited and the possibility that the non-visited had different feeding practices. In his study, Howarth did not make enquiries into the feeding practices of over 500 women because he assumed they would resent enquiry, and it is not clear what formed the basis of his decision (Howarth, 1905, 211). Notes made by health visitors in the data set used in this paper suggests some well-off women were not visited because health visitors assumed they did not need instruction in infant care or because they requested that visits stop. At the other end of the wealth scale, families could feel condescended to and were occasionally rude or physically threatening to health visitors. It is unclear what the balance of such cases was, or whether the feeding methods of unvisited women would have been similar to those of other women. Women coming to infant welfare clinics, where some data were collected, are likely to have been even more self-selected and less typical of the average mother.

A further drawback of these studies is that because they are published, they are naturally limited to the questions that each MOH asked and the aggregate or summary tabulations that he produced. There is no opportunity to examine ‘new’ cross-tabulations, to hold variables constant or to examine paradoxes. For instance, Fildes (1992) found that infant mortality was highest in the London Boroughs where breast-feeding was most widespread, and Howarth (1905) noted that poor sanitation was linked to contamination of the milk supply, but that hand-fed children were generally better housed than breast-fed.

This paper uses data from a similar source to those studies cited above, namely information collected by health visitors regarding feeding methods and mortality, with the difference that the data used here provide information on feeding, survival and other characteristics for each individual child. This detailed, individual level, longitudinal data allows a much more nuanced and rigorous examination of infant feeding and its relationship to socio-economic variables and health, which can sit within and alongside the more general patterns and consequences of feeding in the early twentieth century found by Fildes and Atkins.

## Health visiting in Derbyshire and the data

Under the Notification of Births Acts Local Authorities were encouraged to establish systems to enable health visitors to visit new-born infants as soon as possible after birth and repeatedly thereafter. At least in some places the information gleaned at these visits was recorded in ledgers, some of which still remain. This paper uses one such set for Derbyshire, covering the years 1917–1922. The city of Derby, used in Howarth's Lancet article, had been one of the early pioneers of the notification of births, and most parts of the surrounding county of Derbyshire adopted the 1907 Notification of Births Act. The Act was administered and data was collected by administrative districts: County Boroughs (CBs), Metropolitan Boroughs (MBs), other Urban Districts (UDs) and Rural Districts (RDs). Derbyshire contained one CB (Derby) and four MBs (Buxton, Ilkeston, Glossop and Chesterfield): the data for these was not kept with the data for the rest of the county and has not survived to the present. The dataset therefore consists of the data for 24 urban districts and 15 rural districts, most of which have data from 1917. The areas in the North East of the county around Chesterfield waited until the 1918 Act, so the data for these areas only starts in 1919.^1^

Although the dataset lacks the largest towns in the county, it still encompasses many of Britain's distinctive socio-economic milieux. The urban districts range in size and character from the genteel coaching town of Ashbourne and the spa at Matlock Bath to the lace centre of Long Eaton, the cotton town of New Mills and a significant number of mining towns. Some of the rural areas were also dominated by mining, but the county also contained arable farmland and an upland area of the High Peak. Like other textile areas, those in Derbyshire were characterised by high levels of female employment, low fertility and high infant mortality. The mining areas were also true to form with low levels of female labour force participation, high fertility and high infant mortality. The demographic range in the dataset is therefore similar to that of England at the same time period, but the lack of large towns means that in aggregate its fertility (23 births per 1000 people) was a little higher and its infant mortality (80 infant deaths per 1000 births) was a little lower than the corresponding rates for England and Wales as a whole (21 births per thousand people, and 87 infant deaths per thousand births respectively).^2^

The dataset covers two major social and demographic disruptions: the last two years of the First World War and the 1918–19 influenza pandemic. It is worth a short aside to discuss the possible influences of these on infant mortality and maternal and child health services in Derbyshire. The influenza pandemic killed more people globally than did the war, and its toll was less age- and sex-specific. Unusually for influenza, young adults were particularly affected, but infants and children were not spared and may have been further disadvantaged by the loss of a mother. Pandemic death rates in Derbyshire were very similar to those in the rest of the country (Reid, 2005a). The War probably had a smaller effect on mortality, although it did leave some children permanently or temporarily fatherless (Reid, 2005b).While there were shortages and struggles, far from diverting resources away from maternal and child services, the War focussed attention on the need to preserve and protect the next generation, and there were funding injections from both voluntary and public sources (Dwork, 1987, 211). In this respect Derbyshire was no exception to the rest of the country.

In 1918 the county had 50 health visitors on its books and by 1919 it had achieved full coverage of urban and rural districts. A health visitor visited each birth as soon as possible after notification was received, often in the first week of life, and carried on visiting repeatedly throughout the first year, into the second year and up until the age of about five when children were observed in school. At these visits the health visitors gathered information about the children relating to their health and development. They might have made notes on cards or in their own notebooks, but these were transferred into specially printed ledgers. More information on this data set can be found in Reid, 1999, Reid, 2001a, Reid, 2001b, Reid, 2002.

The ledgers recorded exactly how the child was fed (whether breast-fed, artificially-fed or both) at each visit, together with the date of that visit. For some children early hand-feeding is indicated by the comment ‘A[rtificially] F[ed] from birth’ and sometimes the milk substitute given, for example ‘CM’, ‘C&G’, ‘M&W’, ‘Glaxo & CM’, indicating respectively ‘cows’ milk’, ‘Cow and Gate’, ‘milk and water’, ‘Glaxo and cows' milk’, Cow and Gate and Glaxo being proprietary dried milks. For any child who died, the precise date of death and the medically certified cause were provided by local registrars and added to the ledgers by health visitors. Altogether 51,376 births were recorded over the six year period, of which 93% were live-born and almost all were visited at least once.

Unlike other health visiting programmes, the Derbyshire scheme was intended to be universal, although a small percentage of children were not visited because they could not be found or were deemed not to need visiting (these latter being mainly wealthy families with a private nurse). This introduces a little implicit class bias into the data, but many middle class families were visited repeatedly and there were people from all parts of the social scale who refused visits.^3^ On average, visited infants received 3.5 visits in the first year and a further two in the following year. There was a small amount of active triage, with infants considered to be particularly vulnerable being visited in a more timely manner than others. For example infants identified by midwives as suffering from ophthalmia neonatorum (an eye condition which could lead to blindness if left untreated) were twice as likely to have been visited soon after birth than other infants.^4^ Illegitimate infants, twins, and those whose mothers had died at birth were also visited more rapidly than other infants (Reid, 2001a). In the analysis that follows, being visited before 14 days of age is used as an indication of increased vulnerability.

## Infant feeding patterns

**Fig. 1:**
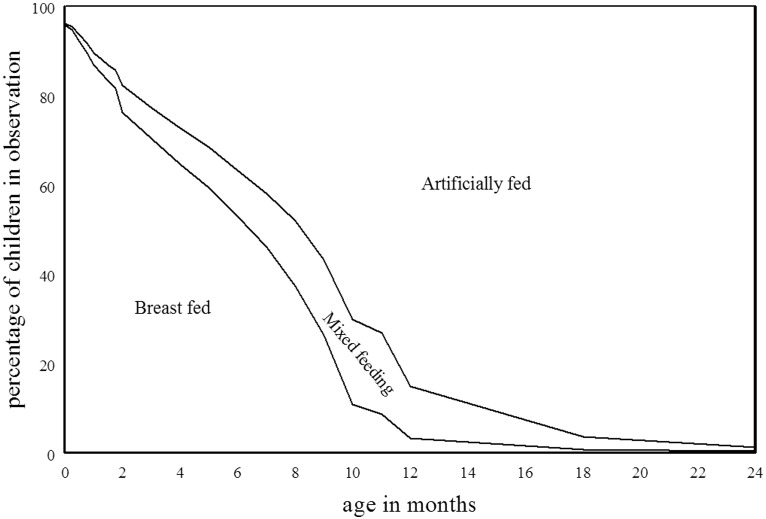
Feeding methods of children in observation in each month.

Source: Derbyshire health visitor data.

Fig. 1 shows the percentages of infants in observation in each month (i.e., whose feeding method is known for that month) described as breast-fed, mixed-fed (i.e., both breast- and artificially-fed), and artificially-fed.^5^ The figure shows that at one month about 86% of children were still exclusively breast-fed, and a further 5% were partially breast-fed; at three months about 70% of children were purely breast-fed and a further 8% were partially so; at six months these figures were 53% and 11% respectively and at nine months only 24% were still apparently exclusively breast-fed and a further 20% were mixed-fed. These figures fall around the middle of the range found by Fildes, who reports a minimum of 76% and a maximum of 92% of babies exclusively breast-fed at one month, in Willesden and Birmingham respectively; with a range of 52% (Tynemouth) to 87% (Bath) breast-fed at three months; 37% (Tynemouth) to 77% (Salford) at six months, and 19% (Tynemouth) to 54% (Wimbledon) at nine months (Fildes, 1998, 257).

Fildes has suggested that although breast-feeding was the ‘norm’ among working classes women in the late nineteenth and early twentieth centuries, it was far from universal, and depended on factors such as recommendations of delivery attendant or other health professional, income and other indicators of status, legitimacy and working patterns among mothers, and ill-health in women (Fildes, 1998, 258–260). It is possible to specifically examine factors such as these using the individual level Derbyshire data, and Table 1 shows the percentages of infants in Derbyshire with different characteristics receiving complete or partial breast-feeding at various ages after birth. Legitimate infants and singletons are not shown as their percentages are very similar to the overall averages.

Illegitimate infants were significantly less likely than legitimate infants to have been breast-fed: at 3 months 20% fewer were receiving any breast milk. Unmarried mothers were more likely to have been working outside the home, and breast-feeding would have been impractical at best and probably considerably problematic. However other working-class women (social classes 3, 4 and 5 whose husbands were skilled, semi-skilled or non-manual workers) were more likely to have breast-fed than wealthier women from social classes 1 and 2, whose husbands were professional and other non-manual workers: the expense and inconvenience of artificial-feeding possibly deterred working-class women from hand-feeding, or notions of prudishness might have discouraged middle-class women from breast-feeding. Those delivered by a doctor were less likely to breast-feed than those delivered by a midwife. This might simply be a correlation with higher social class as middle-class women were more likely to have been delivered by a doctor, but it is also possible that doctors were recommending hand-feeding and possibly pushing their own patent products (Apple, 1995).^6^ Those delivered by both a doctor and a midwife were slightly less likely to breast-feed than those delivered by a doctor only: this group are women for whom there was a problem during birth, hence the need for a doctor to be present in addition to a midwife, and it is likely that exhaustion or illness prevented some of this group from establishing breast-feeding (maternal illness and ‘loss of milk’ being among the most common reasons given for weaning).

There were also demographic factors related to the incidence and duration of exclusive breast-feeding. Twins were the group least likely to have been exclusively breast-fed, but they were more likely to have received mixed-feeding: the mothers of twins who might have found it hard to breast-feed two infants simultaneously clearly supplemented the diet of both. First born children were slightly less likely to have been breast-fed at each age than higher parities. It is possible that this is connected to lower confidence among first time mothers, or it could be the result of a cohort trend towards lower breast-feeding, as in a short-run data series first births are likely to have more recently born mothers than higher parity births.^7^

Many of these factors were correlated: higher social classes were more likely to have been delivered by a doctor, for example, and illegitimate children were mostly first-born. Therefore in order to determine which of these factors were more important, it is necessary to perform a multivariate analysis which permits examination of the effect of each variable while holding others constant.

**Fig. 2:**
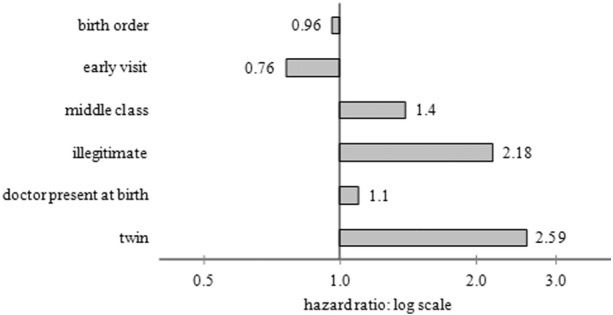
Hazard ratios for the risk of being artificially-fed. Notes: Birth order is a continuous variable; reference categories for the other variables are respectively not early visit (first visit after 14 days), working-class, legitimate, doctor not present at birth (midwife only), singleton birth.

Source: Derbyshire health visitor data, 1917–1922.

Fig. 2 shows the results of a multivariate hazards analysis of the risk of being artificially-fed. A hazards analysis recognises that there is an age pattern to the dependent variable (here the risk of being artificially-fed) and uses observations for individuals at different ages to estimate both the age pattern and the effect of the independent variables on moving that age pattern up or down. In Fig. 2, a value higher than one (a bar to the right of the vertical line) indicates a higher risk of being artificially-fed, and a value below one (a bar to the left of the vertical line) indicates a lower risk of being artificially-fed. The coefficient values are multiplicative, so twins are 2.59 times more likely than singletons to have been artificially-fed at any particular age. The effects are shown in relation to infants in the reference categories, which are legitimate, singleton, first births, delivered by a midwife only, to working-class women, who do not receive an early visit in the first two weeks of life. Only results significant at the 95% confidence level are shown.

The results confirm that twins and illegitimate children were more than twice as likely to have been artificially-fed than singletons and legitimate children. Higher birth order children were still marginally less likely to have been artificially-fed compared to first borns, even when illegitimacy was controlled for. Middle-class infants were 40% more likely, and independently, those delivered by a doctor were 10% more likely to have been artificially-fed. Therefore infants from the middle class who were also delivered by a doctor were 54% more likely to have been artificially-fed (calculated by multiplying the two coefficients together). Other factors are examined but not shown here because they did not have a significant effect on the risk of being artificially-fed include urban or rural location, mining or non-mining location and sex of the child. Finally, this graph shows that infants visited early (within 14 days of birth) were less likely to have been artificially-fed at any particular age. Health visitors were strongly in favour of breast-feeding and they were likely to have encouraged mothers at risk of giving it up (such as these mothers of vulnerable children) to persevere. A leaflet distributed to all new mothers entitled ‘How to rear an infant during the first year of life’ reads as follows:

‘Every infant ought to be fed at the Mother's Breast for the following reasons:

• Because there is not and never can be any nourishment so good as the Mother's own milk.
• Because artificial feeding in the first year is difficult, expensive, troublesome, and dangerous.
• Because an enormous number of children artificially fed die before they are 12 months old of sickness and diarrhoea, and of those who live, many are feeble and unhealthy.
• Because it is easier and healthier for the mother to feed her child at the breast.
• Because the breast-fed child is happy and contented, and the bottle-fed baby is cross and fretful and gives the mother no peace.’

(from leaflet N.B.6. reproduced in Derbyshire MOH report, 1914, p. 12).

One possible reason for hand-feeding, which is very difficult to establish from the data, is the health or condition of the mother. A small proportion of women are thought to be physiologically unable to breast-feed, and others find they cannot sustain it due to illness or exhaustion. The records of some of those who were forced or chose to hand-feed from an early age in Derbyshire were annotated with the reason for giving up breast-feeding, particularly where they were applying for milk substitutes from the council. Typical reasons included: ‘Mother too weak to BF’, ‘Mother too delicate to BF’, ‘No milk’, ‘Br fed at first, mother had influenza 7th week milk failed’, and ‘BM failed during influenza of mother’. Indications of mothers' health were not routinely given for women in the dataset, and can be quite subjective. Those who did not give up breast-feeding did not have any ill-health recorded and it is possible they managed to continue breast-feeding despite ill-health. The influenza pandemic of 1918–1919, however, affords a unique way to look into the effect of ill-health on feeding (see also Reid, 2005a).

**Fig. 3:**
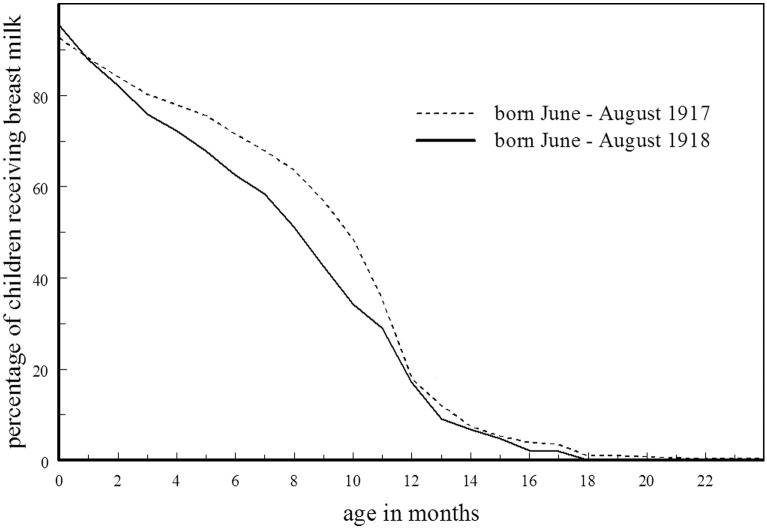
Percentage of infants in observation at each age who were still receiving breast milk: comparing those born in Derbyshire in the summers of 1917 and 1918.

Source: Derbyshire Health Visitor data, 1917–1922.

It is estimated that up to 90% of the population was infected by the influenza pandemic (Johnson, 2006, 117), and although some of these would have been sub-clinical infections it is likely that many new mothers were affected: some will have died but more will have suffered varying degrees of illness. Fig. 3 shows the percentage of infants born June to August 1918 in observation at each age who were still receiving breast-milk: these infants were between 2 and 5 months when the pandemic entered its second and most deadly phase and are likely to have been the hardest hit by any illness experienced by their mothers. As a comparator, it also shows the same percentages for those born in the same months of the previous year when there were no abnormal stressors.

At 6 months there were about 10% more of the 1917 cohort still receiving breast-milk and at 9 months about 15% more. Obviously this does not take into account other influences on feeding, but it is supported by fact that nearly half the monetary value of emergency help given to ‘flu victims in Manchester between 4 Dec. 1918 and 11 Jan. 1919 was in the form of the dried baby milk, Glaxo.

A bout of influenza will have been an acute episode, but poor nutrition and chronic ill-health are likely to have had a similar effect, and this is supported by the fact that chronic conditions such as tuberculosis were also provided as reasons for giving up breast-feeding in Derbyshire. Anecdotal evidence is provided by some of the testimonies sent in 1914 to the Women's Cooperative Guild and published by Margaret Llewelyn Davies (Llewelyn Davies, 1978): one woman wrote ‘I was weak and ill, could not suckle my second baby’ (Letter 136, p.166), another said ‘Before three weeks I had to go out working, washing and cleaning, and so lost my milk and began with the bottle’ (Letter 86, p.111).

## Breast-feeding, hand-feeding and the risks of death

The second of the women quoted above also thanked God that she had not lost any of her children, despite being forced to hand-feed. As the health visitors' leaflet pointed out, and was made clear by Howarth's figures, these children would have been much more likely to die than their breast-fed counterparts. Howarth's calculations classed children as breast-fed or artificially-fed ‘from a very early stage of their existence’ and kept them in that category, but he recognised that this would underestimate the difference in terms of mortality between breast- and hand-feeding, as some children classed as breast-fed would have been switched to artificial-feeding before death. Therefore some of the deaths to ‘breast-fed’ children can be attributed to hand-feeding or mixed-feeding. Changing from hand-feeding to mixed or breast-feeding is virtually impossible so any observed survival advantage among breast-fed infants can only be an under-estimate.

**Fig. 4:**
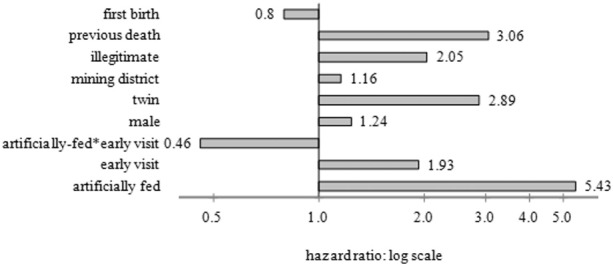
Hazard ratios for the risk of post neonatal mortality, Derbyshire 1917–1922. Notes: Reference categories are respectively: not first birth, mother had no previous child deaths, legitimate, not mining district, singleton, female, not early visit (first visit after 14 days), not artificially-fed. The interaction (artificially-fed*early visit) shows the additional effect of being in both these two categories.

Source: Derbyshire health visitor data, 1917–1922.

A more accurate estimate of the mortality risks will take account of the age at which artificial-feeding was initiated and modern statistical methods allow this. Here time-varying variables are used to move a child from the breast to the artificial-feeding category when they change feeding method, thus creating more accurate measurements of the effect of infant feeding on the risk of death. Fig. 4 shows the results of a multivariate hazards analysis of the risk of post-neonatal mortality (i.e. from the end of the first month of life to the end of the first year), comparing artificial-feeding with other risk factors.

The figure shows that children already artificially-fed were five and a half times more likely than other children to have died between one month and a year, even when other vulnerabilities associated with hand-feeding were controlled, such as being a twin, illegitimate or having a mother who had already experienced a child death. Boys and those in mining districts were also at higher risk of death, but once other variables are controlled first births were slightly less likely to have died. Infants receiving an early visit (within 14 days of birth) were more likely to have died, probably because these were vulnerable infants targeted by health visitors, but the interaction (identifying those both artificially-fed and receiving an early visit, and indicated in the graph above by ‘artifically-fed*early visit’) shows that those visited early, who presumably received information and advice on safe artificial-feeding, were substantially less likely to have died when they became artificially-fed, although because they were artificially-fed they still faced a higher penalty. In more intuitive terms, the predicted post-neonatal mortality for an infant with reference categories of all variables was a mortality rate of 34 per thousand between the end of the first month and the end of the first year. For infants who were otherwise in all reference categories, but were artificially-fed, mortality was 5.43 times this, at 185 per thousand. It was 66 per thousand for those who were visited before fourteen days, but who belonged to the reference categories for each of the other variables. This would predict a mortality rate of 365 per thousand for those in both categories, but the interaction shows that the actual risk was less than half that, and indeed lower than for other artificially-fed children, at 164 per thousand.

In addition to the strong encouragement to breast-feed, the local authority provided advice on artificial-feeding for those who could not continue breast-feeding. Fresh cows' milk was probably the most common breast-milk substitute. It was readily available by the end of the nineteenth century due to the establishment of an infrastructure and middle-men for large scale urban milk supply from rural areas via railways (Atkins, 1992, 209; Beaver, 1973, 250–252).^8^ However doctors and public health officials realised that it contained an inappropriate balance of fats, proteins and sugars for infant diets and mothers were encouraged to modify it to render it more similar in composition to human milk (Coutts, 1911, 30). Precise recommendations varied, but a leaflet circulated by Derbyshire Health Visitors and reprinted in the 1914 MOH Report advised ‘humanising’ cows' milk according to the following instructions:

‘Cow's milk is not like human milk. To make it as much like human milk as possible, stand a glass of milk for 2 or 3 hours with a cover on it until the cream has risen. Pour off the top half of the milk and only use for the baby this ‘top milk’ with the cream in it, sweetened with sugar and diluted with boiled water according to the age of the child. In the first week of life 4 tablespoonsful of top milk to the pint. As the baby grows older each month he will require two tablespoonfuls more of the ‘top milk’ and two tablespoonfuls less boiled water. Each meal should be boiled and cooled immediately before it is given.’ (Derbyshire MOH Report, 1914, 14).

The local authority also supplied free or subsidised milk (mostly dried milk) for those unable to breast-feed and who qualified on the basis of their low income (DCC Maternal and Child Welfare Sub-Committee Minutes, 3rd November 1919; 4th March 1920; Derbyshire MOH Report 1918, 38–9). Much of this was in the form of dried milk, which had recently become popular, with some brands, such as Glaxo, Nestlé, and Cow and Gate, specifically marketed for infant feeding (Coutts, 1918, 3–17; Hudson, 2011). The advantages of dried milk were readily grasped by MOHs; it had a considerable shelf life, was easily transported without the danger of contamination, and could be ‘humanised’ prior to distribution. It became widely distributed by municipal depots and infant consultations, particularly during the war when there was a general shortage of cows' milk. In 1919 Derbyshire County Council spent over four times as much on the provision of milk and virol (an infant food) as on infant welfare centres. The reduced chance of death for those who had particular attention from health visitors suggests the milk substitutes or the advice on feeding given by health visitors were instrumental in reducing the risk of death among artificially-fed infants in Derbyshire, although the risks associated with such behaviour were still high.

## Artificial-feeding and causes of death

**Fig. 5:**
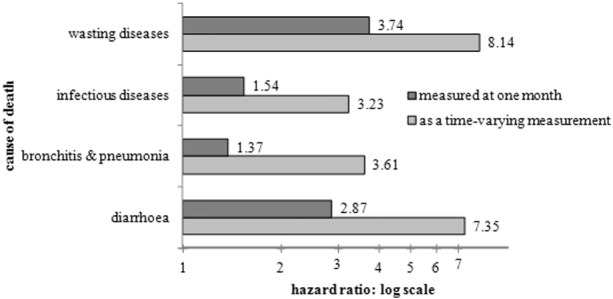
The effect of artificial-feeding on the relative risk of dying from different conditions. Notes: controlling for multiple birth, sex, birth order, previous child death, small house, mining district, urban district, illegitimacy, early visit.

Source: Derbyshire health visitor data, 1917-1922.

Examination of the association of artificial-feeding with the risk of death from particular causes can help to elucidate the ways that hand-feeding disadvantaged children. The analyses shown in Fig. 5 have been run for four selected cause of death groups separately, and children dying from other causes have been censored at the time of death.^9^ Other variables are also controlled, but only the effect of feeding is shown. Two variants of the models have been run – one treating feeding as a time-varying variable and one measuring feeding methods at one month of age. This is because some children will have been changed to artificial-feeding precisely on account of their own ill-health or inability to feed properly: in such cases any death might have been due to the underlying condition of the child rather than as a result of the change to artificial-feeding. Feeding measured at one month will understate the effect of feeding as it will not capture those who started to be artificially-fed after the age of one month, and the time-varying measurement might overstate any causal link from feeding to death due to sickly children being changed to artificial-feeding.^10^ These can therefore be regarded as minimum and maximum effects. The figure shows that artificial-feeding, measured using either method, was associated with higher risks of death from each of the specific groups of causes.

Artificial-feeding is most commonly associated with mortality from diarrhoeal diseases, and the figure confirms that hand-fed children were at a considerably elevated risk from such causes. The mechanism is most likely to have been contamination: infections could have been transferred through improperly sterilised feeding bottles, infected or adulterated milk or from a nearby source of bacterial infection such as a privy midden, a pail closet, an open sewer, or a dung heap (Blackman, 1973, 15–16).^11^ This would have been particularly true in the summer months when high temperatures increased the numbers of the house-flies which were the main vectors of harmful bacteria (Buchanan, 1985, Morgan, 2002). The records do not provide information about water and sanitation in individual houses in Derbyshire, but the MOH reports indicate that water and sanitation varied considerably over the county. Although some places, such as the town of Long Eaton, had virtually eliminated privy middens, on average a quarter of toilet facilities in urban areas and over a third in rural areas were still privies in the late 1920s. Water supply in Derbyshire was generally described by the MOH as satisfactory, but scavenging (the collection of waste, including sanitary waste) was often considered poor. In these circumstances, and without refrigeration facilities, it would have been particularly difficult for many women to maintain the hygiene necessary to prevent any contamination of breast-milk substitutes. The MOH for Derbyshire recognised this in his 1918 report: ‘given all possible instructions in home management it is impossible to bring up healthy children in a house with an unpaved common yard and a privy midden’ (Derbyshire MOH Report, 1918, 38). However the interaction with an early visit was particularly strong for the risk of death from diarrhoeal disease, adding support for the argument that the advice given by health visitors helped mothers to adopt safer hand-feeding practices, even faced with the difficult circumstances of poor sanitation.

Fig. 5 shows that the effect of artificial-feeding on the risk of death was highest, however, for wasting diseases. In an era when breast-milk substitutes did not adequately mimic the dietary composition of breast-milk, artificial-feeding could lead to inadequate growth. This would have applied to home-made substitutes, but also to many of the proprietary and patent foods which had become popular in the late nineteenth century and ranged from small-scale local production to national and widely advertised brands.^12^ Some of these brands were based on dried cows' milk, which may have been mixed with flour. Others were mainly flours, some with malt flour or malt extract and some with treatments or additions which altered the starch in the flour to render it more appropriate for infant digestion.^13^ Most ended up with an excess of protein and carbohydrate and a deficiency of fat in comparison to human milk (Coutts, 1914, 14). The purveyors of many such foods recommended that they should be made up with cows' milk or given alongside milk, but MOHs found that many mothers, particularly the poor who were unable to afford both milk and food, regarded them as an adequate substitute for milk (Coutts, 1914, 31). Coutts' enquiry into proprietary infant foods in 1914 recommended that those containing starch should not be given to infants under seven months of age ‘except on the advice of and under the supervision of a medical man’ (Coutts, 1914, 31). However it is far from clear that medical men were fully aware of the dangers of such inappropriate foods (Apple, 1995).

The higher risks of artificial-feeding for mortality from wasting diseases might therefore be connected to inappropriate milk substitutes, or the inappropriate use of them. However it is important to remember that children who would today be classed as ‘failing to thrive’ might have had their feeding changed in an effort to improve their health. In other words they might have been artificially-fed because they were sickly, not the other way around.

Artificial-feeding was also associated with mortality from infectious diseases (e.g. measles and whooping cough) and from the respiratory diseases which were so often the fatal complications of infectious disease. It is well-established that breast-feeding confers the mother's immunities to some forms of infectious disease, so in an era where such infections were more prevalent, breast-feeding was able to make a real difference to the risk of dying from such causes.

## Conclusions

In conclusion, this exceptionally rich data set has shown that breast-feeding was the norm among working-class British women in the early twentieth century, and that the social gradient was the inverse to that found in Britain today (McAndrew et al., 2012, 30). Middle-class women were less likely to have breast-fed, possibly on the recommendation of their doctor, and women in poor health might have had to switch to artificial-feeding. Other infants less likely to have been breast-fed included twins, illegitimate infants and first births. The dangers of artificial-feeding were already well established in the early twentieth century - at least in terms of the inappropriate nutritional composition of most substitutes in comparison to breast-milk, and in terms of the potential role of infection via contaminated food or feeding receptacles.^14^ Health visitors strongly recommended breast-feeding, and they appear to have been successful in encouraging and supporting breast-feeding among the women they visited particularly soon after birth. However they also provided information and support for hand-feeding safely, and access to suitable artificial food in necessitous cases. The bottle-fed infants of mothers who received early visits from a health visitor were less likely to die than other bottle-fed infants, which suggests that support from health visitors helped women mitigate some of the dangers of hand-feeding. These dangers manifested themselves in enhanced exposure to gastro-intestinal infections and a higher likelihood of dying from diarrhoeal, infectious and respiratory diseases. Artificial-feeding was also linked to higher mortality from wasting diseases, but it is less clear whether this was a product or a cause of artificial-feeding.

Infant feeding, in the promotion of both breast-feeding and safe hand-feeding, were central planks of the campaign to improve infant survival in Derbyshire. This paper does not argue against an interpretation of the Maternal and Child Welfare Movement as an embodiment of ‘a biopolitical regime of governance aiming to administer and optimise life chances’ (Moore, 2013, 55). Nor does it undermine an anatamo-politics of the body which characterises the instruction of women as a means to achieve bio-political goals. The reduction of infant mortality through instruction of women in safe feeding can be justifiably understood in these terms. However this paper is much less supportive of the Foucauldian implication that women are trained ‘to adhere to a pre-existing norm’ (Moore, 2013, 64). Moore portrays this norm as staunchly white middle-class and suggests that ‘any subject positioned outside the concept of normality is pressured by regulation until they fit the norm’ (Moore, 2013, 67). If such a norm existed in the minds of the Derbyshire health visitors, it was a breast-feeding norm, but this paper has shown that breast-feeding was more of a working-class characteristic than a middle-class one. Therefore neither the notion of the middle classes as the norm, nor the working classes as a threat, is supported by the attitudes towards feeding in Derbyshire. Moreover if women could not breast-feed, health visitors would encourage free or subsidised artificial-feeding. Not only were professional, trained health visitors (almost certainly themselves middle-class) pushing a working-class model, but they were also not pushing a single model for mothers, as the concept of normalisation might imply. While biopower can be seen as sinister when it is associated with the assumption that deviance from a class-based or arbitrary norm is dangerous, this analysis shows that this was not a necessary feature of maternal and child welfare programmes, and that biopower is not necessarily sinister.

What can be read into the contrast between the findings of this study and Moore's analysis of the Bolton School for Mothers? Perhaps the most pertinent difference might be that the Derbyshire scheme was local authority run and professionally staffed. In Pooley's study of three contrasting localities the place with strong civic concern and local authority action showed strongest similarities to Derbyshire, whereas the one where the majority of services were delivered by philanthropic middle-class activists was most similar to Moore's philanthropically-run Bolton School for Mothers. Pooley warns against a bias in both contemporary and historiographical interpretations which rely most heavily on localities such as Bromley which contained influential and vocal middle-class individuals, and perhaps the same could be said of local philanthropic institutions (Pooley, 2010, 546). This paper to some extent redresses the balance with an empirical analysis of what health workers actually did and what effect they had on mothers, rather than a study of what filtered through to official reports and newspapers, which is likely to be biased towards middle-class voices.

It is undoubtedly true that it was easier for central and local government to take the ‘motherhood solution’ to the infant mortality problem than to address the structural and political inequalities which condemned large segments of the population to poverty and inadequate facilities. However, contrary to the portrayals of the maternal education solution as undermining the best interests of women and children, this research has shown that the health visitor programme in Derbyshire, and particularly its support for infant feeding, resulted in clear benefits for infant survival. Some of this was through encouragement to breast-feed, which was clearly linked to improved survival chances in a situation where poor sanitary conditions made it difficult to avoid contamination when artificial-feeding. Health visitors also enabled mothers to bottle-feed more safely, although it is not clear whether this operated primarily through instruction in hygiene or through the provision of more appropriate artificial foods. This paper does not argue that health visiting and action regarding infant feeding was responsible for the whole of the infant mortality decline: if it was all due to changes in feeding, the approximate halving of the infant mortality rate in Derbyshire between the 1890s and the end of the War implies levels of breast-feeding at the earlier date which are unfeasibly low. It is also unlikely that the major fall in infant mortality would have been achieved without the gradual improvements in infrastructure and environment which were eventual, if tardy, products of the late nineteenth-century public health reforms. Nevertheless, this paper adds weight to the evidence from Burnley and other places that the infant and child welfare movement did offer concrete benefits (Pooley, 2010; see also Dwork, 1987, Chapter 5; Marks, 1996, 186).

## Figures and Tables

**Table 1:** Percentage of infants not weaned at 1, 3, 6 and 9 months.

	1 month	3 months	6 months	9 months
All infants	87	72	55	41
Illegitimate	73	52	32	21
Delivered by doctor	84	67	50	36
Delivered by doctor and midwife	82	63	49	34
Delivered by midwife only	89	75	58	43
Middle-class	84	66	51	35
Working-class	87	73	56	41
Twins	66	39	22	13
First births	85	67	50	35
Second, third and fourth births	89	75	59	43
Fifth birth and over	87	73	56	42

Note: Legitimate infants and singletons are not shown as their percentages are very similar to those for all infants.

## Footnotes

1 Most previous research using this dataset (Reid, 1999, Reid, 2001a, Reid, 2001b, Reid, 2002) has concentrated the subset of this data excluding the area around Chesterfield.

2 The crude birth rates are for 1921–23, and the infant mortality rates are for 1917–1922 (Registrar General, 1919, Registrar General, 1920a, Registrar General, 1920b, Registrar General, 1922, The Registrar General, 1923a, The Registrar General, 1923b, The Registrar General, 1924).

3 See Reid, 2001a for a more detailed examination of visiting policy by Derbyshire health visitors.

4 Midwives were paid 1 shilling for each notification of opthalmia, in an effort to reduce the condition (Derbyshire County Council Public Health Committee, 14 September 1915).

5 It is assumed that infants were exclusively breast-fed from birth up until the last date when exclusive breast-feeding was mentioned, and that once an infant was recorded as exclusively artificially-fed, they continued to be exclusively artificially-fed from then on. If infants were recorded as breast- and artificially-fed at two different dates it is assumed that they were mixed-fed during the intervening period, but during two visits with different feeding patterns infants are not regarded to be in observation, as it was not known precisely when feeding changed. Because infants tended to be mixed-fed for only a short time, relatively few infants have more than one mixed-feeding observation and the chances of observing them as mixed-fed at a particular month of age is small. Therefore this method is likely to underestimate time spent in the mixed feeding category.

6 ‘Doctor's orders’ was the reason for hand-feeding given for 8% of women for whom reasons were given, and where this excluded the death or desertion of mother. It is unclear why the doctor might have ordered this.

7 Unfortunately the data set does not contain mothers' ages so it is not possible to test this.

8 An alternative, tinned condensed milk had been very popular from the 1870s, and was particularly ill-suited to infant digestion and vulnerable to contamination. The widespread campaign against its use as a food for infants appears to have been successful in Derbyshire (condensed milk was only mentioned in three cases).

9 In other words they have been removed from the analysis at the time of their death so they are treated as being at risk of dying from another cause up until their death, but not after that.

10 Previous analysis has indicated that the effect of weak children being changed to hand-feeding is most concentrated in the first month of life, but persists into the second month (Reid, 2002, 162).

11 The campaign against the particularly dangerous long-tube feeding bottles had already born fruit by the First World War, and most women would have used a boat-shaped bottle which was easier to keep clean (Roberts, 1973, 179–82). Fresh milk might also have been contaminated at the farm or during unrefrigerated transportation. It might also have been adulterated by the addition of chemical preservatives or watered down using unclean water (Atkins, 1992).

12 Proprietary foods used to wean infants according to the Derbyshire records included Allenbury's, which came in numbers 1, 2 and 3 adapted for different ages of child; Virol, a malted food; Neaves; Robinson's Patent Barley; Bengers; Mellins; and Savoury and Moore's.

13 Barley water, which contained a considerable amount of starch, can also be placed in this category.

14 The immunological benefits were also being discovered, although they were probably less well known (Wheeler, Hodgkinson, Prosser, & Davis, 2007).
